# Presynaptic Purinergic Modulation of the Rat Neuro-Muscular Transmission

**DOI:** 10.3390/cimb45100535

**Published:** 2023-10-19

**Authors:** Adel E. Khairullin, Sergey N. Grishin, Ayrat U. Ziganshin

**Affiliations:** 1 Department of Biochemistry, Kazan State Medical University, 420012 Kazan, Russia; khajrulli@yandex.ru; 2 Research Laboratory of Mechanobiology, Kazan Federal University, 420008 Kazan, Russia; 3 Department of Medicinal Physics, Kazan State Medical University, 420012 Kazan, Russia; sgrishin@inbox.ru; 4 Department of Pharmacology, Kazan State Medical University, 420012 Kazan, Russia

**Keywords:** adenosine, A_1_-receptors, A_2A_-receptors, P1-receptors, ATP, P2-receptors, skeletal muscles, rats, synapse, intracellular signaling pathways

## Abstract

ATP, being a well-known universal high-energy compound, plays an important role as a signaling molecule and together with its metabolite adenosine they both attenuate the release of acetylcholine in the neuro-muscular synapse acting through membrane P2 and P1 receptors, respectively. In this work, using a mechanomyographic method, we analyzed the presynaptic mechanisms by which ATP and adenosine can modulate the transduction in the rat *m. soleus* and *m. extensor digitorum longus*. N-ethylmaleimide, a G-protein antagonist, prevents the modulating effects of both ATP and adenosine. The action of ATP is abolished by chelerythrin, a specific phospholipase C inhibitor, while the inhibitory effect of adenosine is slightly increased by Rp-cAMPS, an inhibitor of protein kinase A, and by nitrendipine, a blocker of L-type Ca^2+^ channels. The addition of DPCPX, an A_1_ receptor antagonist, fully prevents the inhibitory action of adenosine in both muscles. Our data indicate that the inhibitory action of ATP involves metabotropic P2Y receptors and is mediated by phospholipase C dependent processes in rat motor neuron terminals. We suggest that the presynaptic effect of adenosine consists of negative and positive actions. The negative action occurs by stimulation of adenosine A_1_ receptors while the positive action is associated with the stimulation of adenosine A_2A_ receptors, activation of protein kinase A and opening of L-type calcium channels. The combined mechanism of the modulating action of ATP and adenosine provides fine tuning of the synapse to fast changing conditions in the skeletal muscles.

## 1. Introduction

The synaptic modulatory function of adenosine-5′-triphosphate (ATP) was discovered at the end of the last century [1,2], and initially it was assumed to be only a precursor of synaptically active adenosine [3]. Subsequently, it was shown that ATP in the synaptic cleft acts independently according to the principle of negative feedback through the ending of motor neurons in the synapses of amphibian phasic muscles [2,3,4]. Released jointly from synaptic vesicles together with the main mediator acetylcholine (ACh) from motor nerve endings, ATP inhibits ACh secretion and muscle contractions [1,2,3,4].

ATP inhibits quantum [1,2,3,4] and non-quantum [5] secretion of ACh from presynaptic endings of motor neurons, acting on P2 receptors, while the end product of its decay, adenosine, implements its action through adenosine receptors [6,7]. Identification of specific subtypes involved adenosine and P2 receptors, and their pharmacological characteristics are the initial stages of studying the effector mechanisms of synaptic regulation by purines of the function of various types of skeletal muscles.

The presynaptic inhibitory effect of adenosine on the amplitude of the evoked phasic endplate currents was established in lake frog muscles [8]. Under similar conditions, the inhibitory presynaptic effect of ATP was demonstrated [9]. At the same time, both pre- and postsynaptic potentiating effects of ATP were established on 2-week-old Xenopus tadpoles and in cell cultures of myocytes [10]. Much less is known about action of purines on the muscles of warm-blooded animals. Experiments on synapses in the rat diaphragm showed a presynaptic potentiating effect of activation of P2 receptors leading to the facilitation of the release of acetylcholine [11], and later the inhibitory effect of adenosine and ATP on end plate potentials was demonstrated at the same object [12]. It is clear that further evaluation of the effects of these purines on other skeletal muscles of warm-blooded animals is needed.

In view of all this, our goal was to investigate the presynaptic transduction mechanisms of ATP and adenosine in the neuromuscular synapse of rat fast and slow skeletal muscles.

## 2. Materials and Methods

The animal study protocol was approved by the Ethics Committee of Kazan State Medical University (protocol code 106/4, from 14 December 2021).

The studies were carried out on the neuromuscular preparations of 38 white Wistar male laboratory rats weighing 140–180 g (4–5 months old), which were kept in groups of 3–5 individuals with unlimited access to water and food. The animals were divided into 8 groups: “Suramin”—6 rats, “8-SPT”—5 rats, “DPCPX”—5 rats, “N-ethylmaleimide”—5 rats, “Rp-cAMP”—5 rats, “Nitrendipine”—5 rats, “Chelerythrin”—4 rats and “d-tubocurarine”—3 rats. Animals were anesthetized by intraperitoneal administration of sodium ethaminal at a dose of 40 mg/kg, bled and *m. soleus* and *m. EDL* were isolated from both hind limbs.

The experimental procedures were made as described earlier in [13]. Briefly, the isolated muscles were fixed vertically tying one end motionless and attaching the other to a mechanical activity sensor and immersed in 10 mL baths filled with Krebs solution of the following composition (in mM): NaCl 118.0, KCl 4.75, CaCl_2_ 2.5, NaHCO_3_ 24.8, KH_2_PO_4_ 1.18, MgSO_4_ 7H_2_O 1.18, glucose 11, pH = 7.4, t = 37 ± 0.5 °C. The thermostat maintains the set temperature. Muscles were stretched with an initial load of 1 g, then left alone for 30 min to adapt to environmental conditions.

Electrical field stimulation (EFS) was performed by stimulating the nerve stump, which was placed in an original design suction electrode. For stimulation, a Digitimer MultiStim D330 stimulator (Digitimer Ltd, Hertfordshire, UK) was used. Muscle contractions were induced by stimulation with rectangular pulses with a frequency of 0.1 Hz, a duration of 0.5 ms, and an amplitude of 10 V for 2 min. Contraction parameters were recorded using a Linton FSG-01 isometric mechanical activity sensor (Linton, UK), and the analog signal was digitized and processed using a Biopack MP100WSW data acquisition system (BIOPAC Systems, Inc., Goleta, CA, USA). The average value of the parameters of all contractions received within 2 min (12 responses) was treated as one result.

After a 30 min initial control, stimulation of the muscle was performed twice with an interval of 5 min, and after ensuring the stability of contractile reactions, experimental procedures were started. All further contractile reactions were calculated as a percentage relative to these initial results.

**Figure 1:**
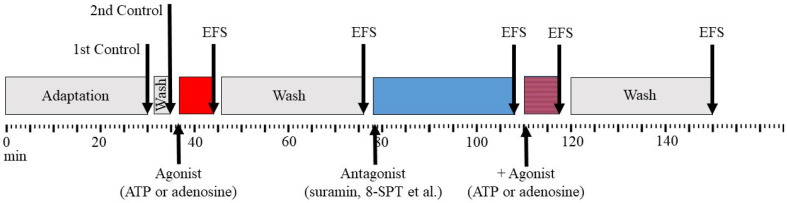
Time line of the experiments.

ATP or adenosine (100 µM) was added to the bath with Krebs solution and the contractile responses of the muscles were evaluated after 7 min. The tissue was then incubated with 8-(*p*-sulfophenyl)-theophylline (8-SPT), 1,3-dipropyl-8-cyclopentylxanthine (DPCPX), suramin, N-ethylmaleimide, 6-(6-aminopurin-9-yl)-2-hydroxy-2-sulfanylidene-4a,6,7,7a-tetrahydro-4H-furo[3,2-d][1,3,2]dioxaphosphinin-7-ol;N,N-diethylethanamine (Rp-cAMPS), chelerythrin, nitrendipine, or d-tubocurarine for 15 min, followed by the addition of ATP or adenosine, and contractile responses were recorded again (Figure 1).

In a separate series of experiments on 3 male rats, the mechanical activity of the muscle was assessed in the presence of d-tubocurarine (a nicotinic cholinoceptor blocker), which was added to baths at a concentration of 10 µM 20 min before stimulation. In another control experiment, muscle preparations were stimulated using the same parameters (rectangular monophasic electrical impulses with a frequency of 0.1 Hz, a duration of 0.5 ms) but with a voltage of 100 V.

Mechanomiographic experiments on rats *m. soleus* and *m. EDL* were analyzed using the SPSS Statistic program. The compliance of the obtained data with the normal distribution was checked using the Kolmogorov’s test. The arithmetic mean of the analyzed parameters and the standard error were calculated. The statistical significance of the observed changes was assessed using Student’s *t*-test for independent and pairwise conjugate samples. Differences were considered significant at *p* < 0.05.

## 3. Results

### 3.1. The Effect of Purines on the Contraction Parameters of Rat m. soleus and m. EDL

**Figure 2:**
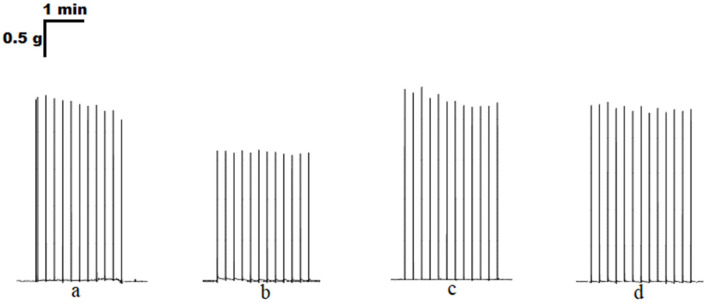
Original traces of contractile responses of the isolated rat *m. soleus* evoked by electrical field stimulation (0.1 Hz, 0.5 ms, 10 V) in control (**a**) and in the presence of ATP (100 μM, (**b**)), suramin (100 μM, (**c**)), a combination of ATP and suramin (both at 100 μM, (**d**)).

**Figure 3:**
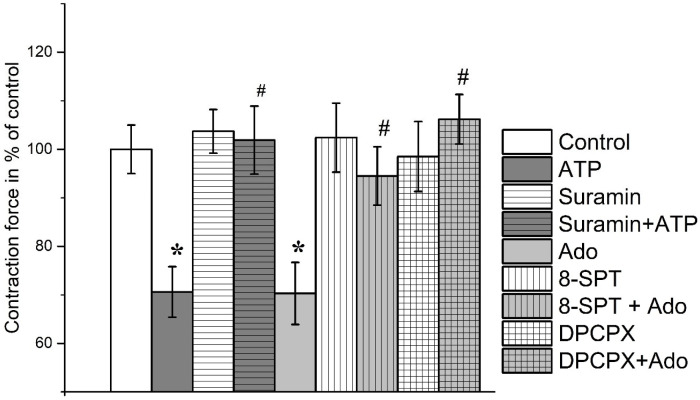
Contractions of rat *m. soleus* evoked by electrical field stimulation in the presence of ATP (100 μM), suramin (100 μM), adenosine (Ado, 100 μM), 8-sulphofenyltheophyllin (8-SPT, 100 μM), DPCPX (0.1 μM). Data shown as mean and standard error, *n* = 8–16. *—*p* < 0.05 vs. controls, #—*p* < 0.05 vs. ATP or Ado alone.

ATP at a concentration of 100 μM inhibited contractions of the soleus muscle induced by EFS to 70.6 ± 5.2% of the initial values (*n* = 18, *p* < 0.05). Preincubation with the P2 receptor antagonist suramin (100 μM) abolished the effect of ATP on the contraction amplitude (101.9 ± 7.0%, *n* = 12, Figure 2 and Figure 3).

Adenosine (100 μM) reduced the amplitude of contractions of the soleus muscle induced by electric field stimulation to 70.3 ± 6.4% (*n* = 16, *p* < 0.05). Additionally, 8-SPT (100 μM), a non-selective adenosine receptor antagonist, almost completely inhibited the effect of adenosine on soleus muscle contractions (94.5 ± 6.0%, *n* = 9, Figure 3).

**Figure 4:**
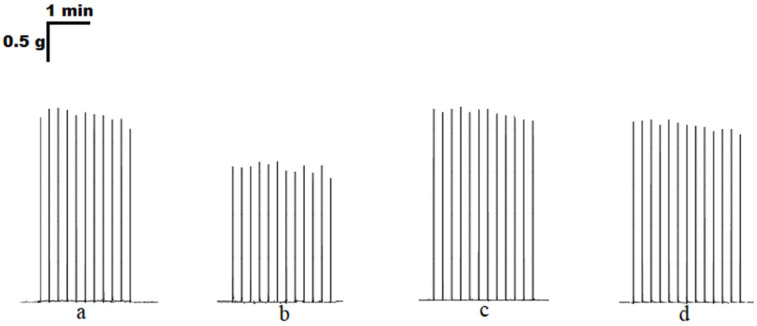
Original traces of contractile responses of the isolated rat *m. soleus* evoked by electrical field stimulation (0.1 Hz, 0.5 ms, 10 V) in the control (**a**) and in the presence of adenosine (100 μM, (**b**)), 8-sulfophenyltheophylline (100 μM, (**c**)), a combination of adenosine and 8-sulfophenyltheophylline (both at 100 μM, (**d**)).

**Figure 5:**
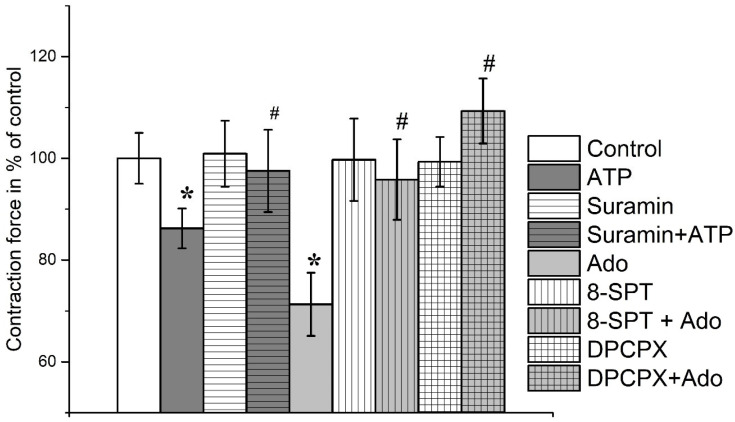
Contractions of rat *m. EDL* evoked by electrical field stimulation in the presence of ATP (100 μM), suramin (100 μM), adenosine (Ado, 100 μM), 8-sulphofenyltheophyllin (8-SPT, 100 μM), DPCPX (0.1 μM). Data shown as mean and standard error, *n* = 8—16. *—*p* < 0.05 vs. controls, #—*p* < 0.05 vs. ATP or Ado alone.

In the presence of ATP (100 μM), the contraction force of *m. EDL* was 86.2 ± 3.9% (*n* = 18), and these data are significantly different from both the initial contractions and the ATP effect in the soleus muscle (Figure 4 and Figure 5).

The inhibitory action of ATP was abolished by suramin (100 μM); in its presence, ATP acted only up to 97.5 ± 8.1% (*n* = 11, *p* > 0.05) of the initial control contractions.

Adenosine (100 μM) inhibited the contraction force of *m. EDL* to 71.3 ± 6.2% (*n* = 16, *p* < 0.05). Additionally, 8-SPT (100 μM) almost completely reversed the effect of adenosine on soleus muscle contractions (95.8 ± 7.9%; *n* = 9, Figure 5).

DPCPX (0.1 μM), a selective A_1_ receptor antagonist, abolished the effects of adenosine on both muscles; the contraction force of the soleus muscle became 106.2 ± 5.1% (*n* = 8, *p* > 0.05) and *m. EDL*—109.3 ± 6.4% (*n* = 8, *p* > 0.05) of the initial control (Figure 3 and Figure 5).

To evaluate whether the EFS parameters used in our experiments induced contractions through nerve stimulation rather than direct muscle stimulation, we tested effects of d-tubocurarine (10 μM), a nicotinic cholinoceptor blocker. We found that d-tubocurarine eliminated all contractile reactions of rat *m. soleus* and *m. EDL* induced by EFS with standard parameters (see Methods). An increase in voltage from 10 V to 100 V led to contractile responses of both *m. soleus* and *m. EDL*, which were not inhibited by d-tubocurarine; the presence of ATP, adenosine, suramin or 8-SPT did not affect these contractions.

### 3.2. Identification of Intracellular Mechanisms Involved in the Effects of Adenosine and ATP

It is known that P2Y receptors and all adenosine receptors belong to the big family of G-protein coupled receptors and some types of these receptors are sensitive to a G-protein blocker N-ethylmaleimide [14].

**Figure 6:**
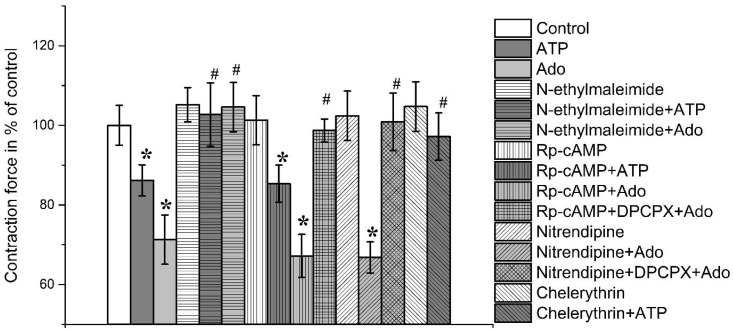
Contractions of rat *m. soleus* evoked by electrical field stimulation in the presence of ATP (100 μM), adenosine (Ado, 100 μM), N-ethylmaleimide (10 μM), Rp-cAMP (50 µM), DPCPX (0.1 μM), nitrendipine (5 μM), chelerythrin (5 μM) or their combination. Data shown as mean and standard error, *n* = 8–16. *—*p* < 0.05 vs. controls, #—*p* < 0.05 vs. ATP or Ado alone.

**Figure 7:**
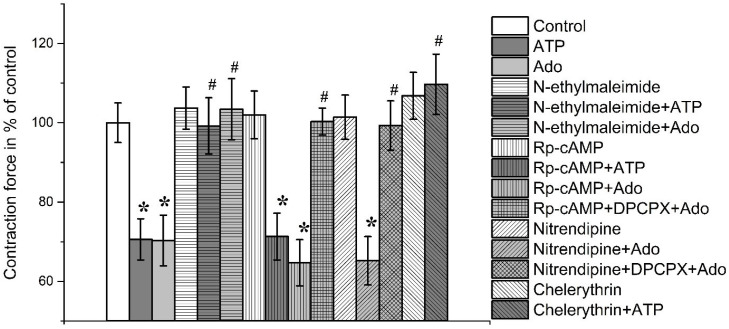
Contractions of rat *m. EDL* evoked by electrical field stimulation in the presence of ATP (100 μM), adenosine (Ado, 100 μM), N-ethylmaleimide (10 μM), Rp-cAMP (50 µM), DPCPX (0.1 μM), nitrendipine (5 μM), chelerythrin (5 μM) or their combination. Data shown as mean and standard error, *n* = 8–16. *—*p* < 0.05 vs. controls, #—*p* < 0.05 vs. ATP or Ado alone.

N-ethylmaleimide at a concentration of 10 μM did not significantly change the force of contraction of both muscles tested. Thus, the force of contraction was 105.2 ± 4.3% (*n* = 8; *p* > 0.05) and 103.7 ± 5.3% (*n* = 8; *p* > 0.05) of the initial control values, respectively, in *m. EDL* and *m. soleus* (Figure 6 and Figure 7).

In the presence of N-ethylmaleimide (10 μM), the inhibitory actions of ATP and adenosine were completely abolished in both muscles. Thus, the strengths of contraction in the presence of ATP (100 μM) were 102.7 ± 8.0% and 99.2 ± 7.1% (*n* = 8, *p* < 0.05), respectively, in *m. EDL* and *m. soleus*. Similarly, in the presence of adenosine, the contractile responses were 103.4 ± 7.7% in *m. EDL* and 104.6 ± 6.2% (*n* = 8; *p* > 0.05) in *m. soleus* (Figure 6 and Figure 7).

These data suggest that the effects of both purines are mediated by a G protein sensitive to N-ethylmaleimide. As such, the involvement of P2Y receptors in the inhibitory action of ATP is confirmed, since only this family of ATP receptors is metabotropic, G protein-mediated.

Possible ways of transmitting the activating signal from the GTP protein are enzyme proteins, the activation of which leads to the formation of various second messengers.

Incubation of the muscle preparations with adenosine-3′,5′-cyclomonophosphoro-thioate (50 µM, Rp-cAMP), a protein kinase A blocker, slightly increased the inhibitory action of adenosine (100 µM), which was 64.7 ± 5.8% (*n* = 8; *p* < 0.05) on soleus muscle and 67.2 ± 5.4% (*n* = 8; *p* < 0.05) on *m. EDL* relative to initial control values (Figure 6 and Figure 7).

In the presence of both DPCPX (0.1 μM) and Rp-cAMP (50 µM), the effect of adenosine on the contraction force was no longer observed, being 100.3 ± 3.4% (*n* = 8; *p* < 0.05) in *m. soleus* and 98.7 ± 2.9% (*n* = 8; *p* < 0.05) in *m. EDL* (Figure 6 and Figure 7).

With incubation of muscle preparations with nitrendipine (5 μM), an L-type calcium channel blocker, the inhibitory effect of adenosine on both muscles was not significantly changed. Thus, contractions of *m. soleus* became 65.2 ± 6.1% (*n* = 8, *p* < 0.05), while *m. EDL*—66.8 ± 3.9% (*n* = 8, *p* < 0.05).

Next, we found out whether the modulating effect of ATP is realized by the intracellular adenylate cyclase metabolic pathway.

In the presence of Rp-cAMP (50 µM), there was no significant change in the inhibitory effects of ATP (100 µM) on both muscles. This suggests that the modulating effects of ATP are mediated independently of protein kinase A.

Chelerythrin, a protein kinase C inhibitor, at a concentration of 5 μM did not significantly change the amplitude of contraction of the studied muscles. Thus, the contraction force of *m. soleus* was 106.8 ± 5.9% (*n* = 7; *p* > 0.05), *m. EDL*—104.7 ± 6.2% (*n* = 7; *p* > 0.05) of the control (Figure 6 and Figure 7).

In the presence of chelerythrin (5 μM), ATP (100 μM) had no effect on the contraction force *m. EDL* (97.2 ± 5.9%, *n* = 8; *p* > 0.05) as well as *m. soleus* (109.7 ± 7.6%, *n* = 8; *p* > 0.05).

## 4. Discussion

As is known, neuromodulation by adenine nucleotides occurs either directly, through the activation of ionotropic P2X/metabotropic P2Y receptors [6,7], or indirectly, after their extracellular breakdown to adenosine and subsequent activation of P1 receptors, which include four different subtypes (A_1_, A_2A_, A_2B_, and A_3_) [15]. Nerve endings, skeletal muscle fibers, and Schwann cells express different subtypes of these purine receptors [16,17,18]. However, the physiological significance of ATP and its metabolites in modulating neuromuscular transmission is far from universally recognized, mainly because studies have been conducted under different experimental conditions in different preparations from a variety of animals. It happened that no attempts were even made to separate the P2 receptor- from adenosine receptor-mediated effects [19].

In this paper, we tried to reveal the mechanisms of the modulation effects of both ATP and adenosine in the synapses of the fast and slow muscles of the rat calf. Initially, we carried out experiments with the application of endogenous ATP. In our experiments, we found that ATP inhibits the contractile activity of the slow soleus muscle of the rat and, to a lesser extent, the fast type of muscle—the extensor digitorum longus. These two types of muscles have certain differences. For example, it was found that they have different isoforms of the sarcoplasmic reticulum calcium pump [20], and that the amount of Ca^2+^ in the sarcoplasmic reticulum of slow muscles is greater than that of fast muscles [21]. The mechanism of the inhibitory effect of ATP may be associated with a decrease in the release of Ca^2+^ from the sarcoplasmic reticulum, as was previously shown for ADP in the slow fibers of the rat soleus muscle [22]. Suramin, a non-selective P2 receptor blocker, abolished this action of ATP. Thus, it can be assumed that ATP exerts its inhibitory effect on rat phasic muscle contractions by influencing P2Y receptors. The participation of presynaptic P2Y receptors in the inhibitory effect of ATP on neuromuscular transmission in the frog sartorius was previously shown [10]. Another confirmation of this assumption is that α,β-methylene-ATP, which predominantly acts on P2X receptors, had no effect on contractions of either fast or slow phase rat skeletal muscles [23].

We also showed that the inhibitory effect of ATP on the quantum yield in amphibian synapses is mediated by P2Y_12_ receptor-associated G_i/o_ proteins sensitive to pertussis toxin [23]. In the experimental data presented in this article, N-ethylmaleimide, a G-protein antagonist, prevents the modulating effect of the purines used, once again confirming the involvement of metabotropic receptors in the inhibitory effect of ATP.

Activation of metabotropic P2Y receptors can trigger an extensive mechanism, which, using the neuromuscular transition in amphibians as an example, includes the production of reactive oxygen species (H_2_O_2_) and prostaglandin E2, which reduces the supply of Ca^2+^ to the frog motor nerve endings [23]. But initially, the activating impulse from the P2Y receptor must be transmitted through the G protein to the primary link of intracellular second messengers, phospholipase or cyclase. In the next series of experiments, we tested the effect of ATP against the background of chelerythrin, a protein kinase C blocker, specific for the intracellular metabolic branch of phospholipase C. It turned out that chelerythrin eliminates the inhibitory effect of ATP on the preparation of both fast and slow muscles. In warm-blooded animals, the involvement of the intracellular phospholipase C metabolic pathway has previously been shown only in diaphragm preparations [23].

The final metabolite of ATP, adenosine, acts through A_1_, A_2A_, A_2B_, or A_3_ receptors. In rodents, A_1_ and A_2A_ receptors show high affinity for adenosine, while A_2B_ and A_3_ are low affinity receptors [15]. The A_1_ and A_3_ receptors are associated with the G_i/o_ protein, while the A_2A_ and A_2B_ are preferably associated with the G_s_ protein. Thus, activation of A_2A_ and A_2B_ typically results in stimulation of adenylate cyclase, resulting in an increase in intracellular 3′-5′-cyclic adenosine monophosphate (cAMP) and subsequent activation of protein kinase A (PKA). Conversely, the activation of A_1_ and A_3_ leads to suppression of adenylate cyclase activity, thus reducing cAMP levels and PKA activation [24]. Remarkably, these receptors can also bind to non-canonical pathways, thus regulating intracellular Ca^2+^ and/or K^+^ concentrations by activating G_q_ protein-coupled mechanisms; others involve direct interference with ionic currents across the plasma membrane through the βγ subunits of G proteins [25,26].

In our experiments, we used N-ethylmaleimide, a G_i/o_ inhibitor, which completely eliminated the inhibitory effect of adenosine. This may mean that the inhibitory effect is mediated by activation of the A_1_ or A_3_ receptor. Continuing the analysis further, we found that in the presence of the A_1_ receptor antagonist DPCPX, the inhibitory effect of adenosine was eliminated.

Previously, it was demonstrated by electrophysiological and neurochemical methods that A_1_-inhibitory and A_2_-excitatory receptors coexist at the rat phrenic nerve terminals to fine-tune the regulation of ACh exocytosis [27]. The experimental data of our group showed that adenosine activates both A_1_ and A_2A_ in non-respiratory skeletal muscle, since the potentiating effect of activation of A_2A_ receptors appeared against the background of DPCPX. This specificity, which is usually observed in the diaphragmatic and central synapses [28,29,30,31,32], may be associated with the proximity of ecto-5′-nucleotidase/CD73 and A_2A_, which contributes to the activation of this subtype of receptors after the formation of adenosine during the breakdown of AMP [28,30].

The A_2A_-mediated facilitation of ACh release from motor nerve endings is thought to be mainly caused by the normally “calm” high-efficiency L-type calcium channels (Cav1) that provide Ca^2+^ influx. When the operation of P/Q-type calcium channels (Cav2.1) prevails, L-type channels are desensitized, for example, during the high-frequency excitation of neurons [32,33,34]. In our experiments, in the presence of L-type calcium channel blockers, the facilitating effect of adenosine disappeared.

The mechanism of subsequent activation of the A_2A_ motor terminal depends on the formation of cAMP by adenylate cyclase [35,36], which leads to stimulation of PKA activity and subsequent Ca^2+^ influx through L-type calcium channels [37,38]. Characteristically, in the results of the experiments presented in this article, the facilitating effect of adenosine continued to be observed against the background of the PKA inhibitor.

Interestingly, in the frog myoneural junction, A_1_ activation inhibits ACh release through a Ca^2+^-independent mechanism, which suggests direct interference with proteins involved in vesicular exocytosis [23]. A decrease in A_2A_ receptor function seems to be a hallmark of aging and some neuromuscular disorders [15].

**Figure 8:**
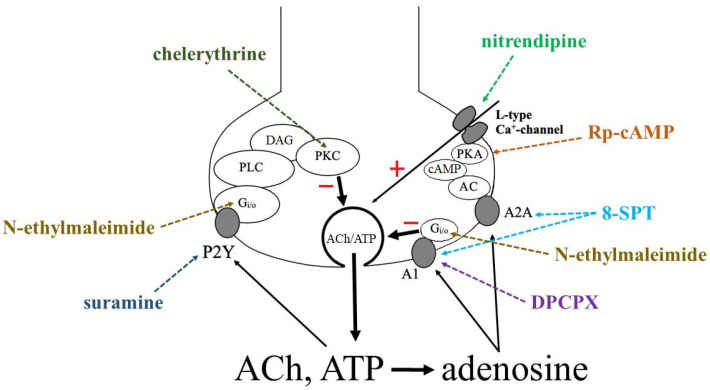
Proposed scheme of purinergic regulation of myoneural transmission of skeletal muscles in warm-blooded animals and the direction of action of the pharmacological agents used in the study. The end of the motor neuron is shown. A synaptic vesicle is presented—a vesicle filled, in addition to the mediator ACh (acetylcholine), ATP. Showing exocytosis of the contents of the vesicles, hydrolysis of ATP to adenosine in the synaptic cleft; activation by adenosine of A_1_—and A_2A_—receptors, and ATP—P2Y—receptors, and their regulation of exocytosis according to the feedback principle through presynaptic intracellular mechanisms. Designations: PKC—protein kinase C, DAG—diacylglycerol, PLC—phospholipase C, PKA—protein kinase A, cAMP—3′-5′-cyclic adenosine monophosphate, AC—adenylate cyclase.

Summing up, in this paper we demonstrated that the two purinergic pathways for modulating the induced ACh exocytosis skillfully complement each other (see Figure 8). It is known that the inhibitory effect of ATP in the synaptic cleft is more often observed at low frequencies of neuronal stimulation (≤1 Hz) [15]. This suggests that it is adenosine that, by activating both presynaptic inhibitory A_1_ and facilitating A_2A_ receptors, depending on the paradigm of the situation, leads the adjusting effects of purines [39,40].

## 5. Conclusions

Thus, we have studied the effects of both ATP and adenosine in the rat fast and slow skeletal muscles. Our data indicate that, in the motor neuron terminals of both studied muscles, the transmission of an activating impulse was mediated by G-protein-coupled ATP receptors (P2Y family) to PLC. We suggest that the presynaptic effect of adenosine consists of two mechanisms: negative and positive, which involve both A_1_ and A_2A_ adenosine receptors. The combined mechanism of the modulating action of ATP and adenosine provides fine tuning of the synapse to fast-changing conditions in the skeletal muscles. The findings reveal potential targets for pharmacological intervention. However, the situation is not yet completely clear, and the purine signaling involved in the above-mentioned effects requires further comprehensive studies.

## Data Availability

Data are contained within the article.

## Abbreviations

8-SPT	8-(p-sulfophenyl)-theophylline
AC	adenylate cyclase.
ACH	acetylcholine
ATP	adenosine-5′-triphosphate
cAMP	3′-5′-cyclic adenosine monophosphate,
DAG	diacylglycerol,
DPCPX	1,3-dipropyl-8-cyclopentylxanthine
EFS	electrical field stimulation
*m. EDL*	extensor digitorum longus;
PKA	protein kinase A,
PKC	protein kinase C,
PLC	phospholipase C,
Rp-cAMPS	6-(6-aminopurin-9-yl)-2-hydroxy-2-sulfanylidene-4a,6,7,7a-tetrahydro-4H-furo[3,2-d][1,3,2]dioxaphosphinin-7-ol;N,N-diethylethanamine
